# Gunshot Wound of the Pregnant Uterus-Operation—Recovery

**Published:** 1891-02

**Authors:** Charles C. Bradley

**Affiliations:** Topeka, Kansas


### GUNSHOT WOUND OF THE PREGNANT UTERUS-OPERATION—RECOVERY.

BY CHARLES C. BRADLEY, M. D., TOPEKA, KANSAS.

Case of Eva M———, age 20, colored, American, single, domestic.

On Sunday, Sept. 7, at 3 p. m., the patient shot herself with suicidal intent, the bullet entering about four inches to the right and on a line with the umbilicus. To complicate matters the girl was about six months pregnant.

When seen she was suffering but sligthly from shock, extremities warm, pulse 100. She was sent to Christ's Hospital.

The history of the case was, briefly, that she had been sitting in a low rocking chair, the shooting being at very close range with a 32-calibre revolver. After consultation by Drs Munn, Alexander, Williamson, and myself an exploratory operation was determined upon.

The patient was chloroformed. The probe at first passed down between the muscles to Poupart's ligament, but upon changing the position of the thighs and relaxing the abdominal muscles the wound was found to be a penetrating one, and an incision made in the median line.

The abdomen was found filled with blood and numerous large clots. Upon examination a bullet wound was found in the right side of the fundus of the uterus, just anterior to the attachment of the right Fallopian tube. This wound was oozing, and was closed by three of Lembert's sutures, checking the hemorrhage completely. A careful examination failed to show any wound of exit in the uterus or any wound of the intestines. The abdomen was carefully cleansed with hot water and closed with silver wire, a drainage tube being first placed in the median line and another in the wound of entrance of the bullet. The operation was performed by Dr. Munn, and was completed at 11:30 Monday morning, September 8th. Patient stood the operation well. Pulse 98, extremities warm.

On Monday, Sept. 8th, at 11 p. m. she threw off a six-months fœtus. Labor easy. The fœtus had a bullet wound just behind the acromion process of the right scapula, another just above the umbilicus, and the right leg was shattered just below the knee.

The temperature was never above 101. The wounds healed by first intention, except just about the wound of entrance of the bullet, the tissues in the vicinity being considerably powder-burnt.

The treatment was salines, opiates as necessary, milk and stimulants. The drainage tubes were removed on the fourth day. The stitches on the eighth day.

The patient went on to recovery, and was discharged from the hospital cured Sunday, Oct. 5, four weeks from date of entrance.

Patient is now employed as a domestic, doing the ordinary work, including washing; has no hernia or other signs of weakening of the abdominal walls. The bullet was not found, but must have passed away with the fœtus or discharges. I am not able to state positively as to this, as she was attended by the nurse in her labor. It has caused no trouble.—North Amer. Prac.

